# *Brucella* induces unfolded protein response and inflammatory response via GntR in alveolar macrophages

**DOI:** 10.18632/oncotarget.23706

**Published:** 2017-12-26

**Authors:** Dong Zhou, Fei-Jie Zhi, Mao-Zhen Qi, Fu-Rong Bai, Guangdong Zhang, Jun-Mei Li, Huan Liu, Hua-Tao Chen, Peng-Fei Lin, Ke-Qiong Tang, Wei Liu, Ya-Ping Jin, Ai-Hua Wang

**Affiliations:** ^1^ Key Laboratory of Animal Biotechnology of The Ministry of Agriculture, Northwest A&F University, Yangling, China; ^2^ College of Veterinary Medicine, Northwest A&F University, Yangling, China

**Keywords:** Brucella. suis S2, goat alveolar macrophages, unfolded protein response, cytokines

## Abstract

*Brucella* is an intracellular bacterium that causes the zoonosis brucellosis worldwide. Alveolar macrophages (AM) constitute the main cell target of inhaled *Brucella*. *Brucella* thwarts immune surveillance and evokes endoplasmic reticulum (ER) stress to replicate in macrophages via virulence factors. The GntR regulators family was concentrated as an important virulence factor in controlling virulence and intracellular survival of *Brucella*. However, the detailed underlying mechanism for the host-pathogen interaction is poorly understood. In this study the BSS2_II0438 mutant (ΔGntR) was constructed. The type IV secretion system (T4SS) virulence factor genes (*VirB2*, *VirB6*, and *VirB8*) were down-expression in ΔGntR. ΔGntR could infect and proliferate to high titers in GAMs without a significant difference compared with the parental strain. ΔGntR infection increased the expression of ER stress marker genes GRP78, ATF6, and PERK in the early stages of its intracellular cycle but decreased the expression of these genes in the late stages. ΔGntR increased greatly the number of *Brucella* CFUs in the inactive ER stress state in GAMs. Meanwhile, ΔGntR infection increased the levels of IFN-γ, IL-1β, and TNF-α, indicating ΔGntR could induce the secretion of inflammatory but not anti-inflammatory cytokines IL-10. Taken together, our results clarified the role of the GntR in *B. suis*. S2 virulence expression and elucidated that GntR is potentially involved in the signaling pathway of the *Brucella*-induced UPR and inflammatory response in GAMs.

## INTRODUCTION

Brucellosis, caused by the genus *Brucella*, is one of the most prevalent zoonotic diseases worldwide with more than 500,000 new people infections annually [1–3]. Brucellosis can be transmitted to human and domestic animals via direct contact with animals, consumption of contaminated dairy products, and inhalation of infected aerosols, etc, among which airborne transmission have been reported to be the most frequent in abattoirs and rural areas [4]. Following inhalation, *Brucella* infects alveolar macrophages (AM) and resides within *Brucella* containing vacuole (BCV) that constitute the intracellular replicative niche [5, 6]. The special replication strategy encourages the bacteria to evade immune surveillance, inhibit host cell apoptosis, complicate vaccine development, which cause a significant economic and health burden [7]. In China, the live attenuated strain *Brucella. suis*. Vaccine strain 2 (*B. suis*. S2) is most widely used vaccine for prevention and control of brucellosis in sheep and other domestic animals [8].

Alveolar macrophages perform different protective functions from other phagocyte during infection. The TNF-α response is different in human or murine AM with monocytes induced by *Mycobacterium tuberculosi*s or heat-killed *Staphylococcus aureus* [9, 10]. AM also differ from other monocytic/macrophagic populations in susceptibility to infection or the intracellular replication kinetics for virus [11, 12]. Interestingly, the anti-inflammatory cytokine IL-10 response is lack by murine AM comparison with peritoneal macro upon lipopolysaccharide exposure [13]. Notably, a recent study showed that *B. abortus* invades and replicates in murine AM and induces significantly increase of TNF-α, IL-1β, IL-6 and IL-12 in AM from different germline mice [4]. However, the interaction of *B. suis*. S2 with the AM has scarcely been studied.

*Brucella* resides within BCVs after phagocytes uptake to avoid degradation within the lysosomal compartments. The BCV undergo maturation and became endosomal BCV (eBCV) with a marker LAMP-1 during the first 8 hr postinfection (pi) [14]. In macrophages, ∼10% BCVs avoid end-stage lysosomes and fuse with the Endoplasmic reticulum (ER) membranes [5]. ER, a large membrane-bound organelle, governs cellular homeostasis by controlling the processing and folding of proteins [15]. During ER fusion, *Brucella* dramatically restructures the ER and disrupts ER homeostasis, inducing ER stress and triggering the unfolded protein response (UPR). In mammals, the UPR is signaled through three sensors that are in the ER membrane: inositol-requiring kinase 1 (IRE1), pancreatic ER eIF2a kinase (PERK), and activating transcription factor 6 (ATF6). In response to ER stress, the binding immunoglobulin protein (GRP78) is recruited away from PERK, IRE1, and ATF6 to promote protein folding capacity. The activation of these ER stress sensors enhances the transcription of chaperones and transcription factor C/EBP-homologous protein (CHOP), which are involved in apoptosis to restore cellular homeostasis. Many studies have shown that the induction of the UPR following *Brucella* infection promotes the intracellular growth of these bacteria [16, 17]. The virulence factors TcpB and VceC induced the UPR in macrophages, supporting *Brucella* intracellular replication [18]. Most recently, the GntR family of regulators was considered as virulence factors that plays a critical role in controlling virulence and the intracellular survival of *B. abortus* [19–21]. Several members of gntR family contribute to pathogenesis of brucellosis [19]. However, the relevance of UPR and GntR virulence in goat alveolar macrophages has not yet been elucidated.

In this study, to test whether gntR has any influence on *B. suis*. S2 virulence, a gntR (BSS2_II0438) mutant ΔGntR was constructed and its virulence was evaluated in dairy goat alveolar macrophages (GAMs). Our results showed that three T4SS structural proteins (virB 2, 6, 8) are down-expressed in the GntR mutant. ΔGntR activated UPR as evident by the increase in GRP78 and CHOP expression. Changes in the UPR influenced the proliferation of *B. suis*. S2 and ΔGntR in GAMs. 4 Phenyl butyric acid (4-PBA), a pharmacologic chaperone that inhibits the UPR, substantially increasing replication and even higher in ΔGntR. Furthermore, ΔGntR induced the secretion of the inflammatory cytokines IFN-γ, TNF-α and IL-1β but not anti-inflammatory cytokine IL-10. These results may reveal a possible mechanism for GntR to be used to decrease *Brucella* virulence and provide information for the further investigation of *Brucella* novel vaccine development.

## RESULTS

### Construction of ΔGntR

**Figure 1:**
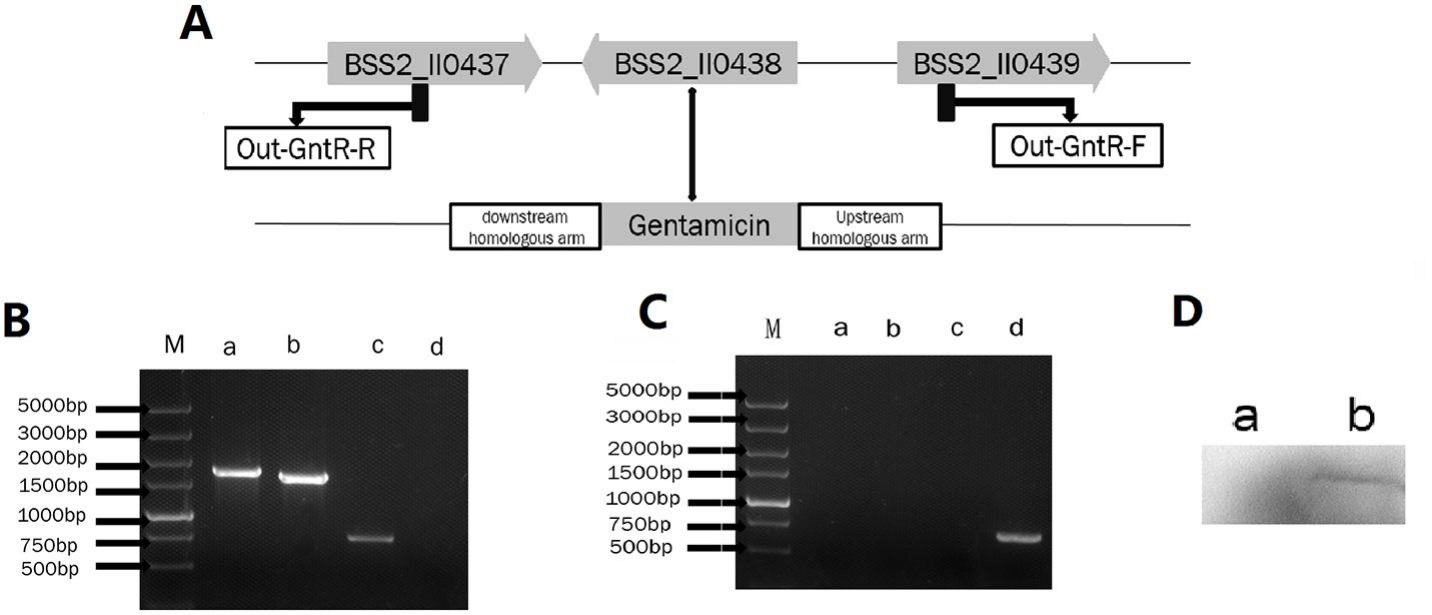
Identification of the ΔGntR strain **(A)** Schematic of the construction of the ΔGntR strain. **(B)** Identification of the GntR gene deletion by PCR amplification in the ΔGntR strain. Lane M: DNA marker DL5000. Lane a: combination of the GntR upstream gene and gentamicin gene. Lane b: combination of the GntR downstream gene and gentamicin gene. Lane c: gentamicin gene. Lane d: GntR gene. **(C)** Identification of the GntR gene by PCR amplification in the *B. suis* strain. S2. Lane a: combination of the GntR upstream gene and gentamicin gene. Lane b: combination of the GntR downstream gene and gentamicin gene. Lane c: gentamicin gene. Lane d: GntR gene. **(D)** Western blotting confirmed the expression of the GntR gene in the ΔGntR strain. Lane a: ΔGntR strain; Lane b: *B. suis*. S2 strain (positive control).

The GntR gene deletion mutant (ΔGntR) based on *B. suis*. S2 was successfully construction. ΔGntR was verified by PCR amplification (Figure 1B and 1C). The results showed that the GntR gene was not amplified in the strain ΔGntR. However, the GntR gene was amplified in the strain *B. suis*. S2. Next, qRT-PCR was performed to quantify the expression of GntR. The results indicated GntR was not transcribed in ΔGntR (date not shown). In addition, the lack of expression of GntR in ΔGntR was verified by Western blotting (Figure 1D).

### GntR regulates the expression of T4SS

**Figure 2:**
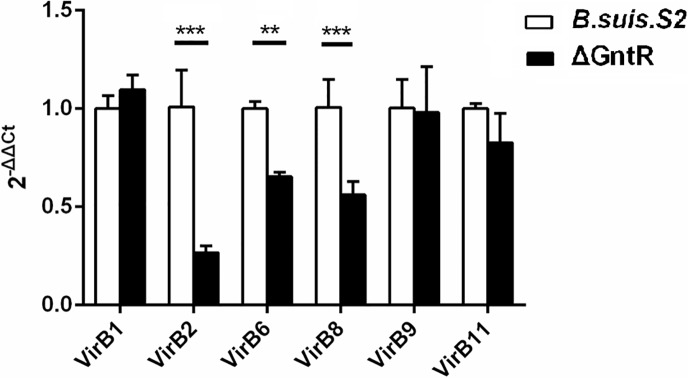
Transcriptional level of T4SS genes obtained by qRT-PCR in the *B. suis*. S2 and ΔGntR strains Strains *B. suis*. S2 and ΔGntR were collected at the exponential phase and resuspended in Trizol for RNA purification. VirB1, VirB2, VirB6, VirB8, VirB9, VirB11 gene expression was detected by quantitative real-time PCR with normalization to 16sRNA. Data are presented as the means ± standard error for triplicate infection samples. ^**^P < 0.01, ^***^P < 0.001.

To investigate the regulatory role of GntR on the T4SS, 6 genes related to T4SS were chosen for the qRT-PCR assay. As shown in Figure 2, compared with *B. suis*. S2, three genes (vir B2, 6, 8) were decreased, and three genes (vir B1, 9, 11) showed no significant difference. These results deduces that T4SS-related genes are among the most GntR-regulated genes.

### ΔGntR infects and replicates in GAMs

**Figure 3:**
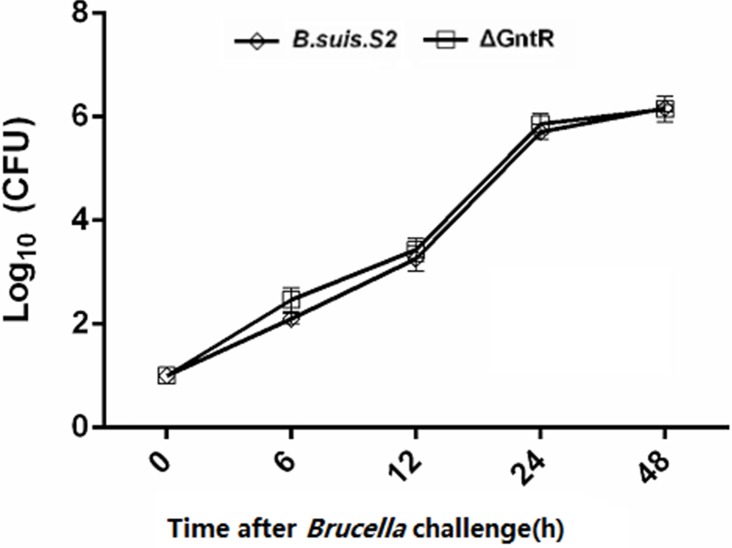
Intracellular survival within GAMs of the *B. suis*. S2 and ΔGntR strains The GAMs were infected with wild type (*B. suis*. S2, rhombus) or GntR mutant *Brucella* (ΔGntR, square). At different times following infection, cell was lysed and CFU determined by transfer to dilution plates. Error bars depict standard deviations of quadruplicate determinations. The data are shown as the means ± standard error from 3 independent experiments.

**Figure 4:**
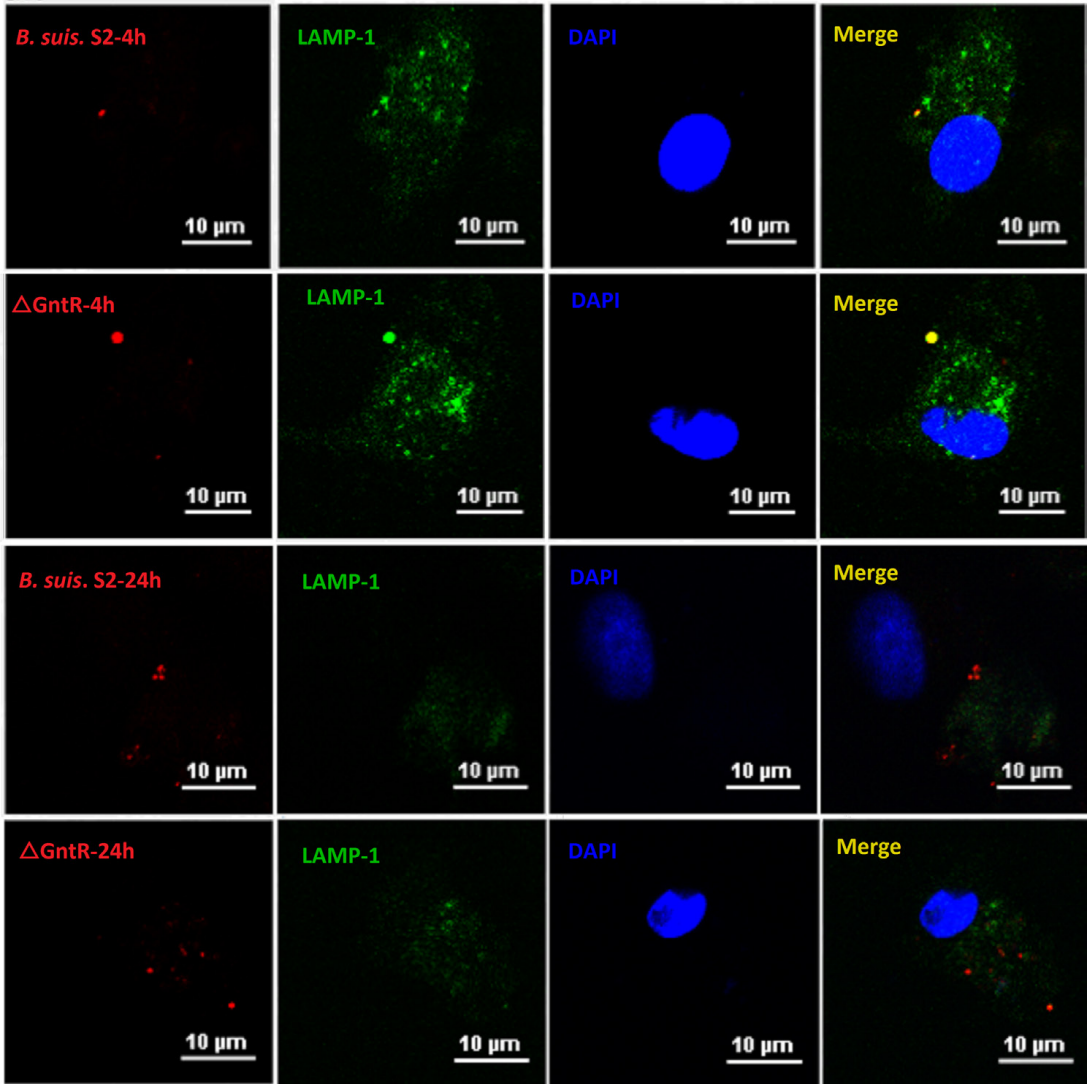
Intracellular survival and traffic of mutant strain ΔGntR in GAM cells Representative confocal micrographs of GAMs infected with *B. suis*. S2 (red) and ΔGntR (red) and immunostained for LAMP-1 (green) at 4 and 24 hr post infection. The data shown are representative of 4–5 independent experiments.

GAMs were chosen (Supplementary Figure 1) and infected with *B. suis*. S2 or ΔGntR at 100 MOI to evaluate whether the GntR gene was involved in bacterial intracellular survival and adherence. The bacterial adherence capacity of ΔGntR was not significantly different (data not shown), and the bacterial intracellular survival of ΔGntR was also not significantly different (Figure 3). To determine whether the ΔGntR strain could efficiently exclude eBCV for intracellular trafficking within AM cells, we measured the number of LAMP-1-positive eBCV at 0 and 24 h hpi. The results showed that the number of LAMP-1-positive eBCV was not significantly different (Figure 4). These results were consistent with the bacterial intracellular survival.

### The ΔGntR mutant triggers ER stress

**Figure 5:**
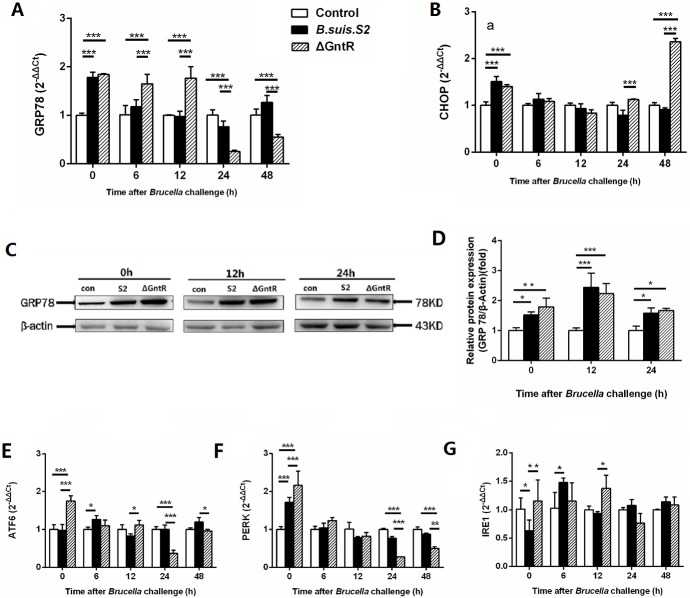
Comparison of ER stress in the *B. suis*. S2-infected and ΔGntR-infected strains in GAMs The mRNA expression levels of GRP78 **(A)**, CHOP **(B)** ATF6 **(E)**, PERK **(F)**, IRE1 **(G)** in GAMs was analyzed using quantitative real-time PCR. GAMs collected after *B. suis*. S2 or ΔGntR (MOI = 100:1) challenge at 0, 6, 12, 24 and 48 h. Data are presented as the mean ± standard error ^**^P < 0.01, ^***^P < 0.001. **(C)** GAMs were infected with 100 MOI of *B. suis*. S2 or ΔGntR for 0, 12 and 24 h, followed by lysis and detection of GRP78 expression by Western blotting. **(D)** Quantification of band intensities was determined by densitometric analysis. The data shown are representative of 3 independent experiments.

To investigate whether GntR was involved in ER stress, GAMs were infected with *B. suis*. S2 or ΔGntR, and the ER stress marker molecules (GRP78 and CHOP) were analyzed by qRT-PCR. The mRNA expression of GRP78 was increased after ΔGntR mutant infection at 0, 6 and 12 h post-infection and was decreased at 24 and 48 h compared with the uninfected and *B. suis*. S2 groups (Figure 5A).

Western blotting results showed that the expression of GRP78 was more strongly in the *B. suis*. S2 and ΔGntR mutant infection groups than in the uninfected group at 0, 12, and 24 h post infection. In line with the mRNA expression, GRP78 protein expression was enhanced in the ΔGntR mutant infection group and *B. suis*. S2 group compared with that in control group at 0,12 and 24 h (Figure 5C, 5D). CHOP mRNA expression in the ΔGntR group was elevated compared with that in the uninfected group at 0 and 48 h (Figure 5B). These results showed that GntR is involved directly or indirectly in ERS and triggered UPR.

To further investigate the UPR pathway during *Brucella* infection, the activation of three UPR sensors was detected by qRT-PCR after infection with *B. suis*. S2 or ΔGntR. The expression of PERK mRNA was increased in both the *B. suis*. S2- and ΔGntR-infected groups compared with that in the uninfected group at 0 h, and the PERK mRNA expression was even higher in the ΔGntR group than in the *B. suis*. S2 group at the same time point. However, 24 and 48 h after *Brucella* challenge, a significant decrease in PERK mRNA expression was observed in the ΔGntR group compared with that in the *B. suis*. S2-infected and uninfected group (Figure 5F). Similar to the expression of PERK mRNA, ATF6 gene expression in the ΔGntR mutant group was elevated at 0 h but decreased at 24 and 48 h compared with the *B. suis*. S2-infected or CONT group (Figure 5E).

### Changing ER stress affects ΔGntR intracellular growth in GAMs *in vitro*

**Figure 6:**
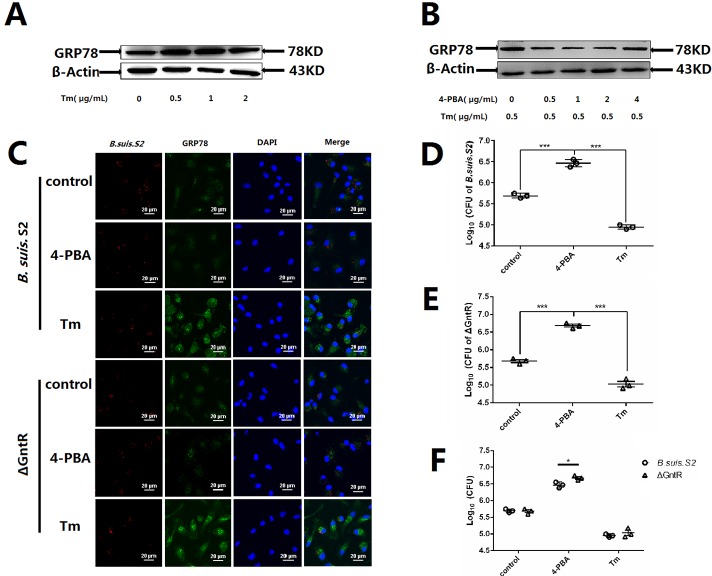
Changing ER stress influences the *B. suis*. S2 and ΔGntR intracellular survival To establish ER stress activated and inhibited model, the UPR marker GRP78 in GAMs treated with different concentrations of **(A)** Tm or **(B)** 4-PBA was analyzed using Western-blot. The data shown are representative of 3 independent experiments. **(C)** Representative confocal micrographs of GRP78 protein (green) in GAMs infected with *B. suis*. S2 (red) or ΔGntR (red) only or plus 0.5 μg/mL Tm or 1 μM 4-PBA at 24 h. The data shown are representative of 4–5 independent experiments. **(D-F)** GAMs were infected with 100 MOI *B. suis*. S2 (D) and ΔGntR (E). Next, 0.5 μg/mL Tm or 1 μM 4-PBA was added before infection. Cells were lysed after 24 h of *B. suis*. S2 infection. CFUs were determined by transfer to dilution plates. CFU numbers are shown on a log_10_ scale. The data represent the means ±standard error deviations from 3 independent experiments and were analyzed by one-way ANOVA. ^***^P < 0.001.

To more directly assess the role ER stress in *Brucella* replication in GAMs, the cells were treated with Tm (ER stress activator) and 4-PBA (ER stress antagonist) to induce or inhibit ER stress. As shown in Figure 6A and 6B, 0.5 μg/ml Tm and 1 μg/ml 4-PBA were used for the further experiments. Inducing ER stress with 0.5 μg/ml Tm significantly increased GRP78 protein expression and inhibited the proliferation of *B. suis*. S2 and ΔGntR at 24 h post-infection compared with that in untreated infected GAMs (Figure 6C, 6D and 6E). Decreasing ER stress with 1 μg/ml 4-PBA significantly inhibited GRP78 protein expression and promoted *B. suis*. S2 and ΔGntR proliferation at 24 h post-infection compared with untreated infected GAMs (Figure 6C, 6D and 6E). In addition, the proliferation of ΔGntR was increased significantly compared with that of *B. suis*. S2 under ER stress inhibition (Figure 6E).

### ΔGntR reduces the production of inflammatory cytokines in macrophages

**Figure 7:**
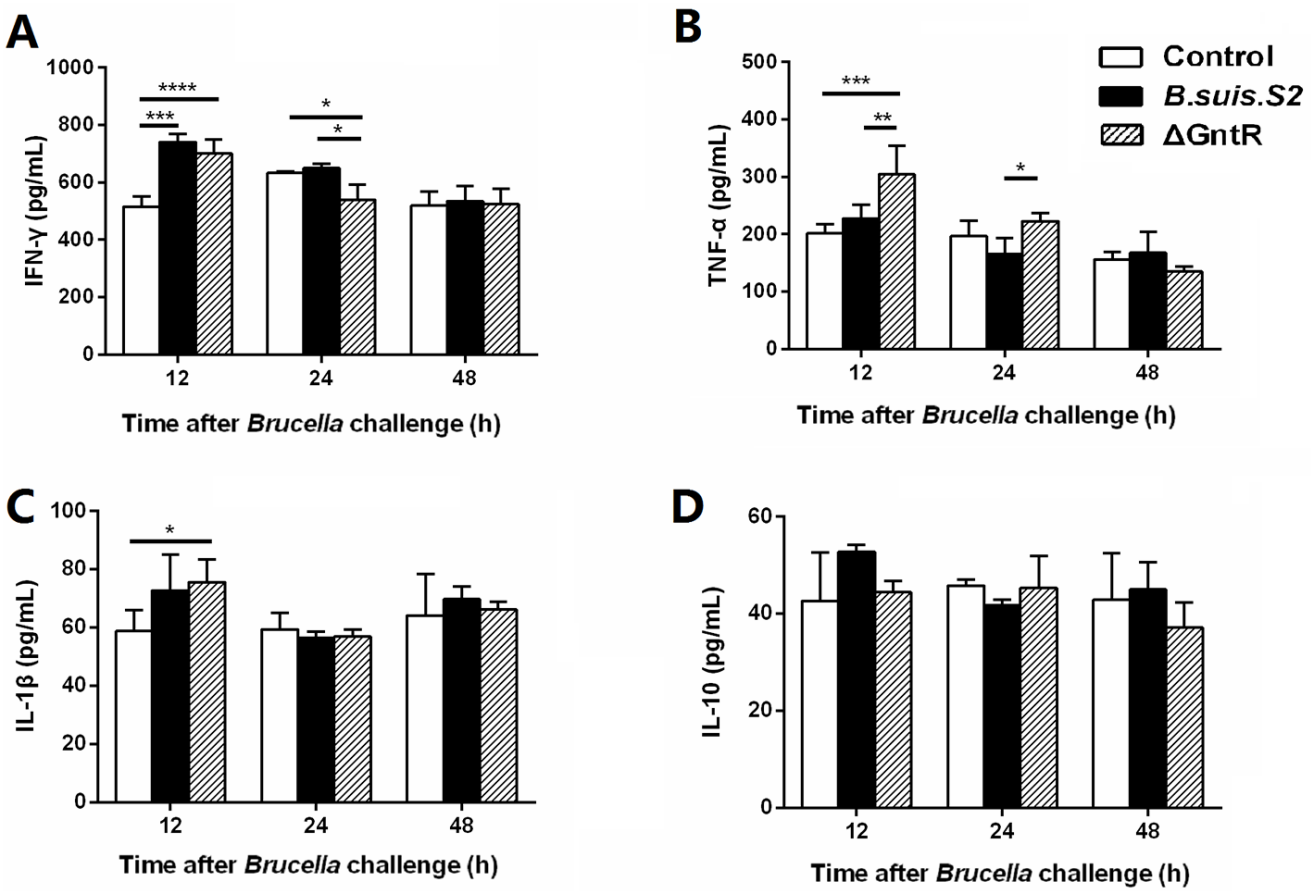
Production of inflammatory cytokines in the *B. suis*. S2- and ΔGntR-infected macrophages GAMs were infected with *B. suis*. S2 and ΔGntR at an MOI of 100. At 12, 24 and 48 h post infection, supernatant samples were collected, and the IFN-γ **(A)**, TNF-α **(B)**, IL-1β **(C)** and IL-10 **(D)** secretion levels were assayed by ELISA. The data represent the means ±standard errors and were analyzed by two-way ANOVA. ^*^P < 0.05, ^**^P≤0.01, ^***^P≤0.001.

To determine the effects of GntR on the secretion of inflammatory cytokines by the infected cells, we collected the supernatant from GAMs infected with *B. suis*. S2 and ΔGntR at 12, 24 and 48h, and measured the secretion levels of inflammatory cytokines IFN-γ, TNF-α, IL-1β and IL-10 by ELISA. The supernatant from GAMs infected with ΔGntR produced higher amounts of TNF-α than those from the *B. suis*. S2-infected and uninfected groups at 12 h, and remained higher than those from the *B. suis*. S2-infected group at 24 h (Figure 7B). The supernatant concentration of IFN-γ was elevated in the ΔGntR- and *B. suis*. S2-infected group compared with that in the uninfected group at 12 h after challenge. However, The IFN-γ production level was lower in ΔGntR-infected GAMs than in *B. suis*. S2-infected and uninfected GAMs at 24 h (Figure 7A). Notably, at 12 h after challenge, the secretion levels of IL-1β were higher in the ΔGntR group than in the uninfected group (Figure 7C). No difference in the IL-10 production level was observed among the 3 groups (Figure 7D). These results indicate that ΔGntR mutant could induce the secretion of inflammatory cytokines but not that of anti-inflammatory cytokines.

## DISCUSSION

Brucellosis is one of the highly contagious zoonotic diseases worldwide with more than 500,000 new people infections annually. Airborne transmission has long been regarded as an important way to infect human and may be also involved in farm animal cases [22]. Alveolar macrophages (AM) is the first phagocytic cells contacted by inhaled of aerosols contaminated with *Brucella*. However, the interaction of *B. suis*. S2 with the goat AM has scarcely been studied. Here, we extrapolate that the *B. suis*. S2 could replicate and induce an unfolded protein response in goat AM.

Bacterial transcriptional regulators regulate various virulence genes at the transcription initiation step in response to cellular signals. The GntR family is one of the most abundant and widely distributed transcriptional regulators families. More than 8,500 GntR family members have been annotated in diverse bacterial genomes and several members of GntR family in *Brucella* have been proved to be involved in the virulence of the pathogen [19, 23]. However, the function of the most predicted GntR members in *B. suis*. S2 is unknown. Our study found that predicted GntR transcription regulator BSS2_II0438 is associated with the expression of T4SS structural genes (*VirB2*, *VirB6*, *VirB8*), Which was consistent with previous studies [19, 24]. VirBs are essential for *Brucella* intracellular replication, modulation of host immune functions, and establishment of chronic brucellosis [1, 25].

Upon phagocytosis, *Brucella* reside within *Brucella*-containing vacuoles (BCVs), which undergo interactions with early endosomes and late endosomes in a controlled manner and partially fuse with lysosomes to become endosomal BCV (eBCVs). In this step, approximately 90% of *Brucella* are degraded, and the remaining 10% survive [26]. Previous studies have shown that *B. abortus* invades and replicates in murine AM, which did not induce cytotoxicity. AM could constitute a durable replicative niche for *Brucella* in infection [4]. Our results show that *B. suis*. S2 replicates in goat AM. deletion of BSS2_II0438 GntR of *B. suis*. S2 did not affect the intracellular survival stage of the bacteria's cycle. However, VirB2 mutant reduced the replication competence of *Brucella* in the J774 macrophages cell line [27]. This phenomenon partly because ΔGntR could not efficiently evade fusion with lysosomes in GAMs. Studies have shown that AM differ from other monocytic/macrophagic populations in the intracellular replication kinetics for certain pathogens [11, 12] These results deduce that the BSS2_II043 GntR protein is not evolved in the induction of eBCV by *B. suis*. S2 or that it may work redundantly with other, as-yet unidentified, effectors to elicit this stage. eBCVs are converted into replication-permissive organelles (rBCVs) via the accretion of ER-derived membranes and exclusion of endosomal membranes. rBCVs can interact with the ER and promote *Brucella* replication. *Brucella* fuses with the endoplasmic reticulum (ER) and provokes an ER stress response called the unfolded protein response. The unfolded protein response (UPR) induced by viruses and bacteria is crucial for their colonization, replication and persistent infection in infected cells. GRP78, as a monitor of ER stress, constrains the biology and life cycles of *Brucella*. Our previous study showed that enhancing the expression of GRP78 promoted the proliferation of *B. suis*. S2, and inhibiting GRP78 protein expression greatly diminished the numbers of *B. suis*. S2 in GTC lines. In addition, altered ER Stress with 4-PBA or Tm affects the intracellular growth of *B. suis*. S2 in GTCs [17]. This finding is in line with our results that enhancing GRP78 protein expression with Tm inhibited the proliferation of *Brucella* in GAMs. Moreover, inhibiting GRP78 protein expression with 4-PBA increased the number of *Brucella* CFUs in GAMs. *B. abortus* exploits VceC to interact with GRP78 and localizes to the ER in HeLa cells [27]. The apparent effect of TUDCA in *Brucella* inducing GRP78 expression is caused by the greatly diminished numbers of bacteria [28]. Increasing GRP78 expression promotes the proliferation of *B. suis*. S2 in GTCs by inhibiting the apoptosis of *B. suis*. S2-infected GTCs [17]. Notably, our results showed that ΔGntR increased greatly the number of *Brucella* CFUs in the inactive ER stress state compared with *B. suis*. S2 in GAMs. These results imply that GntR is potentially involved in ER stress and triggers the UPR via the T4SS of *B. suis*. S2 or other ways.

Inflammatory response is beneficial for host to protection against bacteria invasion [29]. In the early stages of brucellosis, *Brucella* change the cytokine level of IFN-γ, TNF-α, and IL-10 [30]. Recent study clarify that *Brucella* have capability to inhibit the secretion of IFN-γ and increase the secretion of IL-10 that enhances chronic infection [31]. In the present study, we found that goat AMs respond to *Brucella* infection with an increased secretion of IFN-γ in the early stages of infection but no effect on secretion of TNF-α, IL-1β and IL-10. IFN-γ-mediated type I immune responses are essential for clearance of *Brucella* [32]. The significant production of IFN-γ by *Brucella* infected goat AMs deduces that GAMs contribute to the clearance of *Brucella* during aerosols infection. Interestingly, we found that ΔGntR-infected GAMs resulted in increased secretion of TNF-α, IL-1β and IFN-γ. Previous study has shown that mouse alveolar macrophages response to *Brucella* infection with a marked increase in secretion of TNF-α and IL-1β [4]. TNF-α controls macrophage activation and neutrophils recruitment to the infected site. the expression of TNF-α enhances the elimination effect on *Brucella* by reinforcement bactericidal and antigen-presenting functions of macrophages [33, 34]. The significant production of TNF-α by ΔGntR-infected GAMs suggests that BSS2_II043 GntR protein is essential for *Brucella* to evade the host elimination. Consistent with previous study, *Brucella*-infected murine AMs resulted in increased expression of IL-1β, but the effect of IL-1β for the response to inhaled *Brucella* had not been explored [4]. The anti-inflammatory cytokine, IL-10 was not elicited by both *Brucella* and ΔGntR infection. Study indicated that lack of production of IL-10 resulted in an increased ability of mice to control *B. abortus* infection. The UPR has increasingly been shown to associated with inflammatory response. Activation of IRE1 by ER stress can activate the mitogen-activated protein kinase (MAPK), JNK, which is a key player in the response to inflammatory stimuli [35]. Macrophages undergoing ER stress induction enhanced IL-1β, IL-6 and TNF-α production in response to LPS stimulation *in vitro* [36]. In *Brucella*-infected macrophages, VceC can activate IRE1-dependent secretion of IL-6 and TNF [27]. Although residing in the ER might promote the survival of *Brucella*, it is possible that UPR induction might function as an innate immune mechanism against invading bacteria. Take these results extrapolated that BSS2_II043 GntR protein is essential for *B. suis*. S2 survival in goat AMs. Further studies are needed to clarify the mechanisms of the BSS2_II043 GntR protein involved in regulating the inflammatory response *in vivo*.

**Figure 8:**
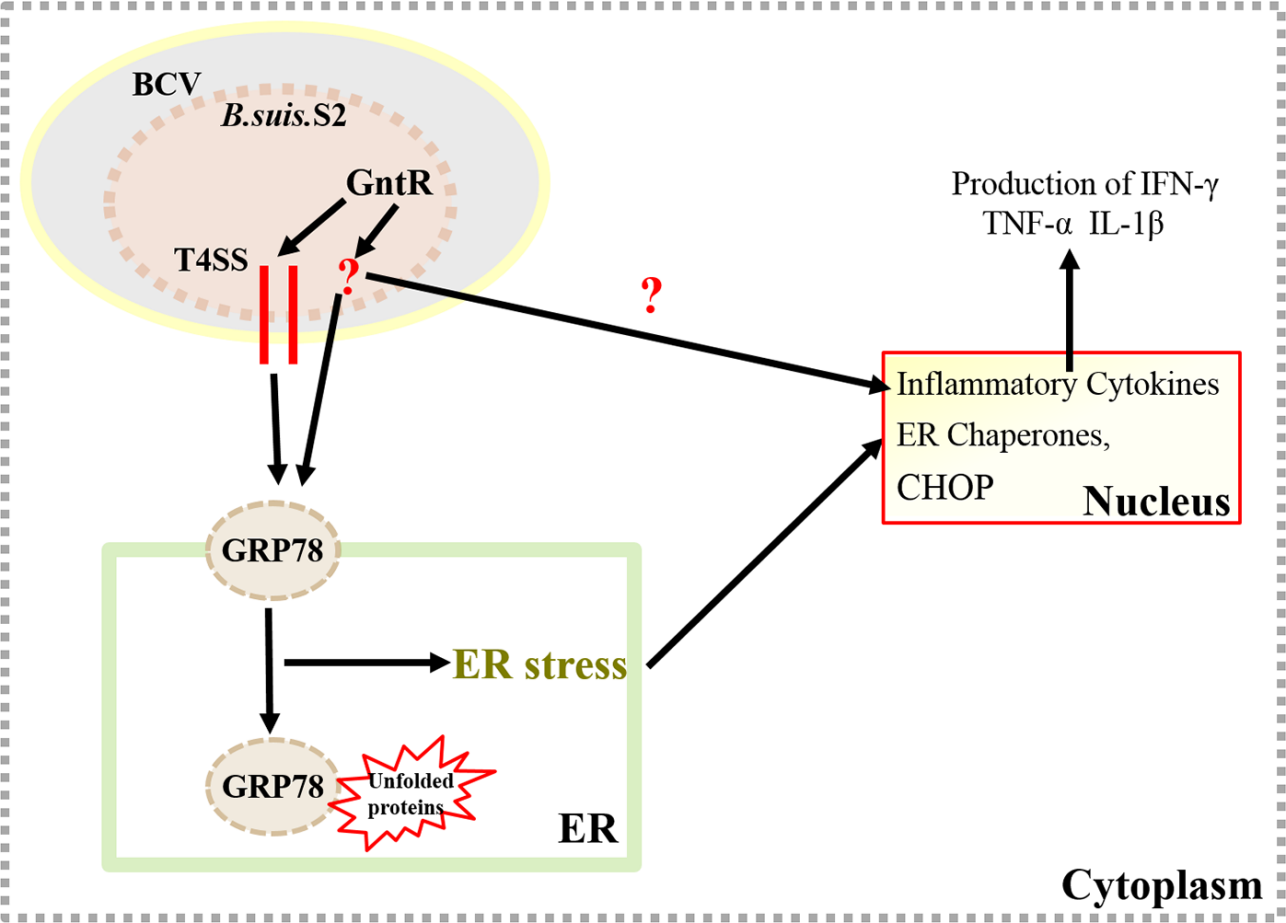
Update model of the GAMs response to *B. suis*. S2 infection The GntR-like transcription regulator BSS2_II0438 can decrease the T4SS genes expression of B. suis. S2, which is involved in ERS pathway and the inflammatory response in GAMs.

In this study, our results provide novel evidence that the GntR-like transcription regulator BSS2_II0438 can decrease the T4SS genes expression of B. suis. S2, which is involved in ERS pathway and the inflammatory response of GAMs (Figure 8). Our results may provide new insights into understanding the interaction between rBCV and ER during *Brucella* infections. The findings will also provide new targets to prevent and control *Brucella* infections.

## MATERIALS AND METHODS

### Ethics statement

The sampling procedures were compliance with the “Guidelines on Ethical Treatment of Experimental Animals” (2006) No. 398 established by the Ministry of Science and Technology, China.

All animals were treated in strict accordance with the “Guidelines on Ethical Treatment of Experimental Animals” (2006) No. 398 from the Ministry of Science and Technology, China. The sampling procedures in the present study had received prior approval from the Experimental Animal Manage Committee of Northwest A&F Universit with the approval license number was 2017ZX08008005.

### Bacterial strains and cells

*B. suis*. S2 cells were obtained from the Chinese Veterinary Culture Collection Center (Beijing, China). *B. suis*. S2 was cultured in tryptic soy broth (TSB) or tryptic soy ager (TSA, Takana). The number of *B. suis*. S2 cells was counted by plating on TSA. This *Escherichia coli* strain DH5α (Takana) was grown on Luria-Bertani medium. When appropriate, 50 μg/mL ampicillin, kanamycin and gentamicin were respectively added. Plasmid PUC19 was purchased from Takana.

Goat alveolar macrophages (GAMs) were collected by bronchial lavage from the lungs of healthy 5-month-old Guanzhong dairy goats. Donor animals were determined to be *Brucella* free. GAMs were cultured in RPMI-1640 medium (HyClone) supplemented with 15% fetal bovine serum (FBS, Gibco) at 37°C with 5% CO_2_.

### Construction of the mutant strain ΔGntR

ΔGntR was constructed as described previously [37]. Briefly, primers for construction were designed using the sequence GntR in the *B. suis*. S2 genome and PBBR1MCS-5. The 607-bp upstream fragment, 825-bp downstream fragment and 751-bp gentamicin fragment were obtained in three independent PCR reactions using primer STAR Max Mix with primer pairs GntR-UF/GntR-UR, GntR-DF/GntR-DR and G-F/G-R. After purification by gel extraction, the three fragments were cloned into PMD19T-simple and then were digested with *Pst*1 and *Xba*I, *Sac*I and *EcoR*I, *Xba*I and *Sac*I sequentially and then subcloned into the *Xba*I- and *Sac*I-digested PUC19 plasmid. The recombinant plasmid with the correct sequence was designated PUC19-GntR and was introduced into DH5α. *B. suis*. S2 was electroporated with PUC19-GntR. The potential GntR deletion mutant ΔGntR was selected by plating on TSA-containing gentamicin, which was then verified by PCR, quantitative real-time PCR (qRT-PCR) and Western blotting. Primers were designed according to the strain *B. suis*. S2 genome in Table 1.

**Table 1:** Primers used in the study

Primer	Accession no	Sequence	Product (bp)
GntR-UF	CP006962.1	CTGCAGAACCGCTTCAGCCATTCTTCCA	607
GntR-UR		TCTAGATATCGTTCAGATACATAAAGCTCGG	
GntR-DF	CP006962.1	GAGCTCAGCGGCCCATATCGG	825
GntR-DR		GAATTCATCGGGCTTTCCGCAGT	
G-F	EF153731.1	TCTAGATTGACATAAGCCTGTTCGGTTCGTA	751
G-R		GAGCTCTTAGGTGGCGGTACTTGGGTCGATA	
Out-GntR-F	CP006962.1	AGCATGGAACAGAACGTCATAATCA	
Out-GntR-R		TGCAAGGAAGGAACGGATT	
RT-GntF	CP006962.1	GCACTACAGGCAGGACACG	280
RT-GntR		TGAGGCTCAACCCGCTAA	
RT-virB1-F	CP006962.1	TGCCATTTCTTGTCCTCG	143
RT-virB1-R		AAGGGAAACGGGTTGACG	
RT-virB2-F	CP006962.1	CGCTGCAATCGAGCCTAA	154
RT-virB2-R		GTGCCGGAATGCCATCTT	
RT-virB6-F	CP006962.1	ATATCAGCACCTATTCGGAGTG	154
RT-virB6-R		ATGGCTGCGATGTTCCAC	
RT-virB8-F	CP006962.1	GACAAGCAATATGGCTCAAA	193
RT-virB8-R		CCGATTCCGACATCAAAGA	
RT-virB9-F	CP006962.1	ATTTACGCAAGGCTGGGAGT	216
RT-virB9-R		TGAACACGATAGGCAACA	
RT-virB11-F	CP006962.1	CCGCAAGCCGTCTTTCAC	297
RT-virB11-R		CGATCACCCTTTCAAGCTGTAC	
RT-16sRNA-F	CP006962.1	GTGGAATTCCGAGTGTAGAGG	371
RT-16sRNA-R		GTCCAGCCTAACTGAAGGATAG	
RT-CHOP-F	AY943948.1	AGGACCACCAGAGGTCACAC	193
RT-CHOP-R		TGCCACTTTCCTTTCGTTTT	
RT-GRP78-F	DQ029323.1	TGAAACTGTGGGAGGTGTCA	171
RT-GRP78-R		TCGAAAGTTCCCAGAAGGTG	
RT-PERK-F	XM_005686691.1	CCCCATCCGCTACTGAACG	151
RT-PERK-R		GGGCTGCTGGAGTGTCTTG	
RT-IRE1-F	XM_005694366.1	ACTCCCTCAACATCGTTCACAG	208
RT-IRE1-R		CTCCTTGCAGTCTTCGCTCA	
RT-ATF6-F	AY942654	AACCAGTCCTTGCTGTTGCT	204
RT-ATF6-R		CTTCTTCTTGCGGGACTGAC	
RT-GAPDH-F	XM_005680968.1	GGCGCCAAGAGGGTCAT	100
RT-GAPDH-R		GTGGTTCACGCCCATCACA	

### Cell infection assay

Cell infection was performed as previously reported [17]. Briefly, GAMs were seeded in six-well plates (2 × 10^6^ cells per well) and were infected with *B. suis*. S2 and ΔGntR at 100:1 MOI. After 4 h of incubation, the GAMs were washed three times with PBS and then further cultured with cell culture medium containing 50 μg/mL kanamycin to eliminate *B. suis*. S2 and ΔGntR adhering to the GAMs and in the culture medium. After 1 h, the GAMs were washed three times with PBS and were further cultured with cell culture medium containing 25 μg/mL kanamycin to avert continuous infection. This time was considered the 0 h and time point of treatment with Tm (ER stress activator) and 4-PBA (ER stress antagonist). The cells were collected, and relevant experiments were performed at specific times (0, 6, 12, 24, and 48 h).

For intracellular survival assays, wells of infected cells at 0, 6, 12, 24 and 48 h were washed and incubated with 500 μL of 0.5% Triton X-100 in PBS for 10 min. The lysates were diluted in PBS and were plated onto TSA at 37°C with 5% CO_2_ for 72 h. The number of bacteria at each time point were determined.

For adherence, wells of infected cells at 0 h were washed and incubated with 500 μl of 0.5% Triton X-100 in PBS for 10 min. Next, the cells were incubated with 500 μL of 0.5% Triton X-100 in PBS for 10 min. The lysates were diluted in PBS and were plated onto TSA to determine the colony-forming units.

### RNA isolation and quantitative real time PCR

Strains *B. suis*. S2 and ΔGntR were collected at the exponential phase. The GAMs of infected *B. suis*. S2 and ΔGntR were collected at 0, 6, 12, 24 and 48 h. Total RNA was extracted using TRIzol (Invitrogen, Inc., Carlsbad, CA, USA). RNA was subjected to reverse transcription using the Vazyme RT Reagent Kit according to the manufacturer's protocols. Next, qRT-PCR was performed using an ABI 7500 Sequencing Detection System and SYBR Premix Ex Taq™. Primers were designed according to the strain *B. suis*. S2 genome in Table 1. The relative transcription levels were calculated using the 2^−ΔΔCt^ method.

### Western-blotting analysis

*B. suis*. S2-infected GAMs were harvested in a tube and then were lysed on ice for 30–45 min in lysis buffer. The supernatant was obtained by centrifugation for 15 min at 14,000 rpm at 4°C. The protein concentration was determined by the BCA assay. Total cellular protein was extracted with 5 × SDS-PAGE loading buffer after boiling for 5 min in water. Samples were electrophoresed on a 12% polyacrylamide gel for SDS-PAGE. The gels were then electro-transferred onto PVDF membranes. The membranes were blocked for 1 h in Tris-buffered saline containing 0.5% Tween-20 (TBST) with 5–10% skimmed milk at room temperature, and then were incubated overnight at 4°C in blocking solution containing rabbit anti mouse GRP78 antibody (Proteintech, 11587-1-AP, 1:1000 dilution) or mouse anti-human β-actin (Proteintech, 60008-1-lg, 1:2000 dilution). The membranes were washed five times with TBST for 5 min and then were incubated for 1 h with the goat anti rabbit IgG or goat anti mouse IgG antibody conjugated to HRP (Invitrogen, 31460, 31430, 1:5000dilution). Finally, the membranes were washed five times in TBST for 5 min. The blots were visualized using the Gel Image System (Tannon, Biotech, Shanghai, China).

### Immunofluorescence assay

GAMs were seed on 15-mm glass diameter coverslips in 24-well plates and were infected with *B. suis*. S2 and ΔGntR at 100 MOI. Immunofluorescence staining of LAMP-1 and GRP78 in *B. suis*. S2 was performed. At 0 and 24 h pi, infected cells were washed twice with PBS and then were fixed with 4% paraformaldehyde at room temperature for 30 min. After three washes with PBS, cells were incubated with PBS containing 0.25% Triton X-100 at room temperature for 20 min. After three washes with PBS, goat anti-brucella polyclonal antibody (1:100 dilution), rabbit anti- mouse GRP78 monoclonal antibody (Proteintech, 11587-1-AP, 1:200 dilution) and mouse anti-LAMP-1 monoclonal antibody (Biolegend, 121601, 1:200 dilution) were used as the primary antibody. Donkey anti-goat alexa fluor 555, Donkey anti-mouse alexa fluor 488 (Life technologies, 1736967) and Donkey anti-rabbit alexa fluor 488 (Life technologies, 1796365) were used as the secondary antibody at 1:200 dilutions. Next, coverslips were mounted on glass slides, and cells were observed under a microscope. Assays were performed in triplicate.

### ELISA

GAMs cultured in 6-well plates were infected with *B. suis*. S2 or ΔGntR at an MOI of 100:1 in triplicate wells. At 12, 24 and 48 h post infection, IL-1β (Biomatik, EKC32210), IL-10 (Biomatik, EKU05174), IFN-γ (Novetinbio, BG-GT11412) and TNF-α (Biomatik, EKU07943) production in the culture supernatants was tested using an ELISA kit according to the manufacturer's instructions.

### Statistical analysis

Statistical analysis was performed using Graphpad Prism software 6 (GraphPad software Inc., La Jolla, CA, USA). Statistical significance was determined using two-way ANOVA or one-way ANOVA. *P* values less than 0.05 were considered statistically significant.

## SUPPLEMENTARY MATERIALS FIGURE



## Abbreviations

AM: Alveolar macrophages
ER: endoplasmic reticulum
*B. suis*. S2: *Brucella suis* vaccine strain 2
T4SS: type IV secretion system
GAMs: goat alveolar macrophages
IFN-γ: interferon-γ
IL-1β: interleukin-1β
tumor necrosis factor-α: TNF-α
BCV: *Brucella* containing vacuole
UPR: unfolded protein response
IRE1: inositol-requiring kinase 1
PERK: pancreatic ER eIF2a kinase
ATF6: activating transcription factor 6
CHOP: C/EBP-homologous protein
4-PBA: 4 Phenyl butyric acid
