# Supplementary material for: Brucella induces unfolded protein response and inflammatory response via GntR in alveolar macrophages
Source: Oncotarget. 2017 Dec 26;9(4):5184–96. doi: 10.18632/oncotarget.23706 (PMC5797042; doi:10.18632/oncotarget.23706)
Supplement: Supplementary file 1 [file oncotarget-09-5184-s001.pdf]

Brucella induces unfolded protein response and inflammatory response via GntR in alveolar macrophages

SUPPLEMENTARY MATERIALS

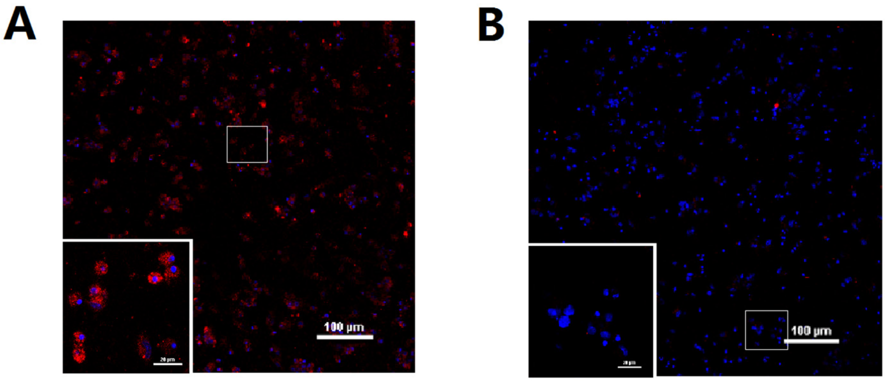

Supplementary Figure 1: Identification of GAMs by CD14 Immunofluorescent staining. (A) The CD14 Immunofluorescent staining in GAMs. The red colour represents CD14 staining, and the blue colour indicates nuclear staining. **(B)** Negative control. The blue colour indicates nuclear staining.
